# Non-standard radiotherapy fractionations delay the time to malignant transformation of low-grade gliomas

**DOI:** 10.1371/journal.pone.0178552

**Published:** 2017-06-01

**Authors:** Araceli Henares-Molina, Sebastien Benzekry, Pedro C. Lara, Marcial García-Rojo, Víctor M. Pérez-García, Alicia Martínez-González

**Affiliations:** 1 Department of Mathematics, University of Castilla-La Mancha, Ciudad Real, Castilla-La Mancha, Spain; 2 INRIA Bordeaux Sud-Ouest, team MONC, Institut de Mathematiques de Bordeaux, Bordeaux, Nouvelle-Aquitaine, France; 3 Department of Radiation Oncology, Negrín Las Palmas University Hospital, Las Palmas GC, Canarias, Spain; 4 Department of Pathology, Hospital de Jerez de la Frontera, Jerez de la Frontera, Cádiz, Spain; Purdue University, UNITED STATES

## Abstract

Grade II gliomas are slowly growing primary brain tumors that affect mostly young patients. Cytotoxic therapies (radiotherapy and/or chemotherapy) are used initially only for patients having a bad prognosis. These therapies are planned following the “maximum dose in minimum time” principle, i. e. the same schedule used for high-grade brain tumors in spite of their very different behavior. These tumors transform after a variable time into high-grade gliomas, which significantly decreases the patient’s life expectancy. In this paper we study mathematical models describing the growth of grade II gliomas in response to radiotherapy. We find that protracted metronomic fractionations, i.e. therapeutical schedules enlarging the time interval between low-dose radiotherapy fractions, may lead to a better tumor control without an increase in toxicity. Other non-standard fractionations such as protracted or hypoprotracted schemes may also be beneficial. The potential survival improvement depends on the tumor’s proliferation rate and can be even of the order of years. A conservative metronomic scheme, still being a suboptimal treatment, delays the time to malignant progression by at least one year when compared to the standard scheme.

## Introduction

Gliomas are the most frequent type of primary brain tumors. Patients diagnosed with gliomas typically die because of the complications related to the disease. No substantial progress has been made in the last decades, thus these types of cancer remain to be a major challenge for medicine.

Low-grade gliomas (LGG) are a subgroup of gliomas (WHO grade II primary brain tumors) usually having slow growth and moderate incidence that are diagnosed mostly in young adults. The median survival time for LGG patients is about 5 years after diagnosis [1, 2]. After a variable time, these tumors undergo the so-called malignant transformation (MT) and progress, to a higher-grade tumor (HGG). After the MT, the neurological symptoms and complications become more difficult to handle and the mean patient’s survival time decreases significantly.

Many LGG patients present few, if any, neurological symptoms for extended periods of time. The use of surgery on diagnosis results in a better outcome and is now the default option in many centers [3–5]. However, the decision on the timing and specific combination of resection, radiation therapy (RT), and/or chemotherapy use on each patient is a complex one. Typically, it is based on the consideration of many variables including age, performance status, and location of tumor [2, 6]. Since LGGs are such a heterogeneous group of tumors with variable natural histories, the risks and benefits of each therapy must be carefully balanced.

In this paper we focus our attention on RT. It is known that RT is beneficial for the patient in terms of survival [7]. It is now well known that immediate RT after surgery increases the progression-free survival, but does not improve overall survival [8]. Although conformal techniques are decreasing the amount of radiation received by the surrounding normal brain tissue this therapy may induce serious long term neurological deficits. Currently, RT is usually offered to patients with a combination of poor risk factors such as age, sub-total resection, diffuse astrocytoma pathology [9], or those suspicious of having a high grade tumor.

Mathematical modeling has the potential to help in finding the optimal timing for radiation therapy and in developing optimal fractionation schemes for selected patient subgroups. Although some studies on non-standard fractionations have been developed in clinical settings [10], they have been very limited. Moreover, the availability of high resolution magnetic resonance images allowing the quantitative measurements of tumor growth rates (and other geometrical imaging biomarkers) may provide key information for the development and validation of such models [11].

Mathematical research on gliomas has been very extensive although much focused on the more frequent HGGs [12–28]. Most of these models are based on the Fisher-Kolmogorov equation [29] to be described in detail later and add different layers of complexity depending on the level of biological detail incorporated into the model. As to RT, it has been studied mathematically both in the context of HGGs [30–35] and LGGs [36–40].

Ribba et al [36] developed a model based on ordinary differential equations describing the response of LGGs to different therapies with a number of undetermined parameters that can be fit to describe the individual patient’s response with a good qualitative agreement. More recently, Pérez-García et al [37] constructed a simple spatial model able to describe the known phenomenology of the response of LGGs to RT including the observations from Pallud et al [41]. An alternative explanation to the phenomenon has been developed by Badoual et al [40] using an oedema-based model. Galochkina et al [38] found that small variations of the standard dose distributions and/or changes in the fractionation led only to minor improvements at best, in agreement with clinical experience.

In clinical practice, radiation doses are given in a very short period of time with the purpose of killing every clonogenic cell without allowing the tumor to regrow between fractions [42]. In fact, the most typical course of RT for LGGs consists of 30 doses of 1.8 Gy given from Monday to Friday for 6 weeks. This is a reasonable practice when radiation therapy is used with curative intent and/or in fast-growing tumors. However, it is not obvious that the optimal fractionation should follow the same scheme when used on a radioresistant tumor. LGGs grow slowly, with a very low number of mitoses seen per field which means that only a small fraction of tumor cells is proliferating at a given time. Typical numbers for LGG are usually around 4% of the tissue showing positivity for proliferation markers as measured by Ki-67/MIB-1 immunostaining [43, 44]. Thus, if only few cells are dividing, it may be reasonable to enlarge the distance between fractions. i.e. to resort to a protracted therapeutical scheme, to allow for more tumor cells to enter in the cell cycle rendering radiation fractions more effective. This idea was explored by Pérez-García and Pérez-Romasanta [39]. The authors fixed the dose per fraction to be the one used in practice but left free the time intervals between fractions. They found a potential improvement in survival by using a protracted regime with dose time inter-spacing between one and two months.

**Fig 1:**
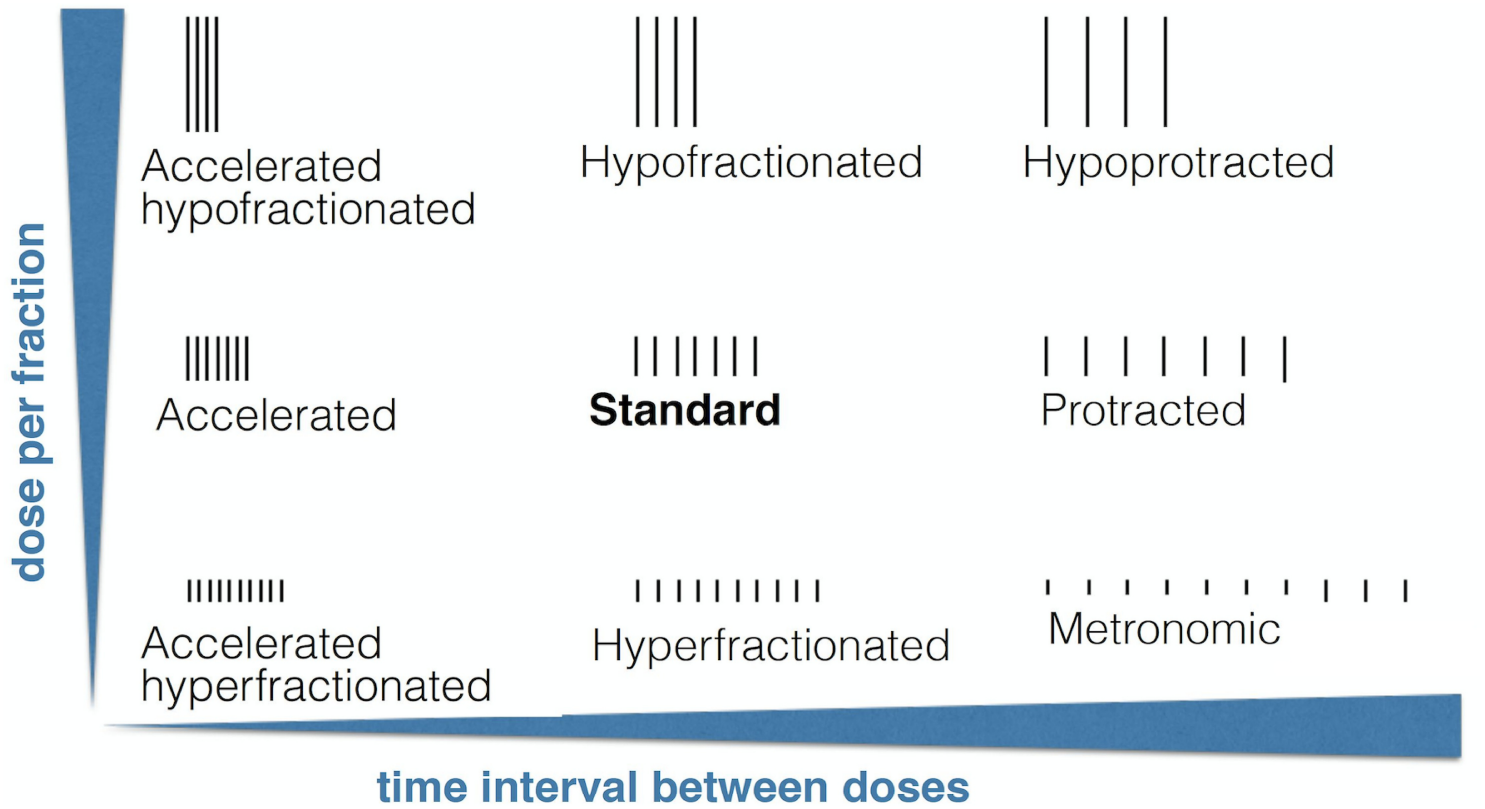
Radio(chemo)-therapy schemes classified by the dose per fraction and the time spacing between fractions. All schemes are defined in relation to the standard one.

To fix notation we will refer to treatment schemes with spacing between fractions smaller/larger than one day as *accelerated/protracted*. Also treatment schemes with dose per fraction smaller or larger than 1.8 Gy are referred to as *hyperfractionated* or *hypofractionated* respectively. The special case of hyperfractionated therapies with time separation between fractions larger than the standard could be denoted as hyperprotracted if we follow the established naming convention. However, it is customary to refer to these protocols as *metronomic*. Fig 1 summarizes different potential schemes to be considered later in this paper.

**Fig 2:**
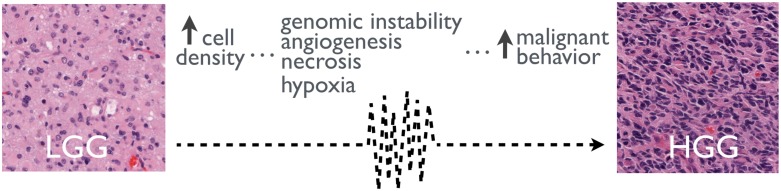
The increase of celularity may lead to the malignant transformation of LGGs. Left and right images are immunohistochemical staining for Hematoxilyn and Eosin for LGG and HGG biopsies respectively.

In this paper we discuss non-standard radiotherapy fractionations potentially deferring the most the time to the malignant transformation (TMT) of the LGG into a HGG. One of the driving forces of the MT may be the increase in mutation rates originated by alterations of the tumor micro-environment. These changes could be driven by the continuous cell density growth that ultimately lead to vessel damage, generation of hypoxic foci, the stabilization of hypoxia dependent signaling molecules such as hypoxia inducible factor-1*α* (HIF-1*α*) and the increase of genomic instability [24, 45–47] (see Fig 2).

To accomplish the task of finding the optimal therapy in this paper we took the dose per fraction, the time between fractions and the number of fractions to be free parameters. We intended the toxicity of the novel schemes to be the same (or lower) than that of the schemes currently in use. Thus, the number of doses was taken to be a function of the chosen toxicity and the dose per fraction. This means that we consider finding novel RT fractionations schemes by changing two independent variables: the dose per fraction and time interval between fractions.

## Materials and methods

### Equations for the evolution of the cell density

The dynamics of tumor cells was described mathematically in this study using the Fisher-Kolmogorov equation [29]

$$\frac{\partial u}{\partial t}=D\nabla^{2}u+\rho \left(1-u\right)u.$$ (1)

Eq (1) accounts for the growth of a spatio-temporal density *u*(*x*, *t*) of tumor cells in units of a maximal cell number. Tumor cells proliferate with a typical time 1/*ρ* and have a characteristic mobility (diffusion) coefficient *D*. This model has been used extensively to describe the dynamics of gliomas and as the basis to construct models of response to RT [33] and specifically for LGGs [39]. Other more complicated models for LGGs keep the same structure for the tumor cell compartment while incorporating a second compartment for a different quantity related to the radiological response, either dying cells [37, 38] or edema [40], that does not affect the response to the therapy.

The model equations are considered in a three dimensional domain Ω of the brain supplemented with initial data $u_{0}\in C^{2}\left(\bar{\Omega }\right)$, and no-flux boundary conditions. It is possible to write an equation for an upper bound for the tumor amplitude *U*(*t*), 0 ≤ *u*(*x*, *t*) ≤ *U*(*t*) that is [39]

$$\frac{dU}{dt}=\rho \left(1-U\right)U$$ (2)

with *U*(0) = *U*_0_ as the initial maximum tumor cell density in the tissue. In addition to providing an upper bound for the tumor cell density. Eq (2) has also been shown to describe the evolution of the tumor amplitude

$$A\left(t\right)=\max_{x\in \Omega }u\left(x,t\right)$$ (3)

with good accuracy in one-dimensional scenarios [37].

### Mathematical description of radiation therapy

To include radiation therapy in the model, we used the classical linear-quadratic (LQ) model [42]. Thus, for a radiation dose *d*_*j*_ given at a time *t*_*j*_, we took the survival fraction *S*_*f*_(*d*_*j*_), i.e. the fraction of cells that are not lethally damaged to be given by

$$S_{f}\left(d_{j}\right)=e^{-\alpha_{t}d_{j}-\beta_{t}d_{j}^{2}}$$ (4)

The parameters *α*_*t*_ and *β*_*t*_ are the linear and quadratic coefficients for tumor cell damage of the LQ model.

Eq (4) does not account for the time dynamics of the tumor and DNA damage during each radiation fraction, thus the effect of RT was assumed to be instantaneous. This is so because RT is given in a time (typically about 10 minutes) that is very short in comparison with typical cellular proliferation times in LGGs [37]. Repair processes occur in the time-scale of minutes or a few hours, which is negligible for the very long evolution times of LGGs.

The full treatment consists of a total dose, *D*, split in a series of *N* radiation fractions with doses per fraction {*d*_*j*_}_*j* = 1, ⋯, *N*_, such that *d*_1_ + ⋯ + *d*_*N*_ = *D*, given at irradiation times {*t*_*j*_}_*j* = 1, ⋯, *N*_. The tumor amplitude *U*(*t*) at the irradiation times satisfies.

$$U\left(t_{j}^{+}\right)=S_{f}\left(d_{j}\right)U\left(t_{j}^{-}\right)$$ (5)

Damage to normal tissue (or toxicity/standard toxicity) caused by RT can be estimated using Eq (4) but with the parameters corresponding to the healthy tissue *α*_*h*_, *β*_*h*_, with *α*_*h*_/*β*_*h*_ ≈ 2 [42]:

$$E\left(N,d_{1},\ldots ,d_{N}\right)=-\ln\left[\prod_{j=1}^{N}S_{f}\left(d_{j}\right)\right]=\alpha_{h}\left(D+\frac{1}{\alpha_{h}/\beta_{h}}\sum_{j=1}^{N}d_{j}^{2}\right)$$ (6)

In addition, acute tissue reactions and other side effects depend: (i) on the total volume irradiated (the so-called volume effect) and (ii) on the maximal dose per fraction d_*_ used. We did not consider in this paper spatial aspects of radiation therapy or other complications and thus will assume that all tumor cells within the tumor receive the same amount of radiation.

### Time to malignant transformation

One of the driving forces of the MT is the increase in mutation rates originated by micro-environmental alterations. These changes are due to the fact that the cell density increase leads to vessel damage, generation of hypoxic foci, stabilization of hypoxia dependent signaling molecules such as HIF-1*α* and an increase in genomic instability [45–47]. Previous studies have reported the role of hypoxia as triggering the MT of oral sub mucous fibrosis [48]. Several mathematical models of gliomas, which are consistent with experimental facts, have also discussed that high density cell foci may lead to the MT of LGGs [22, 24].

Thus, we assumed that the tumor shows LGG features while the maximum tumor density is below a critical level U_*_. On the basis of that we define the TMT as the time when the tumor amplitude (*U*) reaches the critical threshold (U_*_).

### Optimization problem

In this paper our goal was to choose the therapy in order to make TMT as large as possible when the dose per fraction, the number of fractions and the time between them are treated as free parameters restricted to a constant toxicity. We considered the dose per fraction and the time between fractions to be fixed during the full radiation course, that is *d*_*j*_ = *d* and *t*_*j*_ − *t*_*j*−1_ = Δ, ∀_*j*_ ∈ {1, …, *N*}, where *N* is the total number of fractions. In that case, we computed explicitly the TMT using the exact solution of Eq (2) and the recursion formula Eq (5), thus TMT is obtained to be

$$\text{TMT}\left(N,\Delta ,d\right)=\underset{treatment\;time}{\underset{︸}{N\Delta }}+\underset{regrowth\;time}{\underset{︸}{\frac{1}{\rho }\log\left[\frac{U_{*}\left(1-U_{N}\left(d\right)\right)}{U_{N}\left(d\right)\left(1-U_{*}\right)}\right]}}$$ (7)

where *U*_*N*_ is the tumor amplitude at time NΔ (end of treatment) given by

$$U_{N}\left(d\right)=\frac{U_{0}{\left(\alpha S_{f}\left(d\right)\right)}^{N}}{1+U_{0}\left(\alpha -1\right)\left[\frac{{\left(\alpha S_{f}\left(d\right)\right)}^{N}-1}{\alpha S_{f}\left(d\right)-1}\right]}$$ (8)

and *α* = exp(*ρN*), provided *U*_*N*_/*S*_*f*_ ≤ U_*_. This restriction is necessary to ensure that *U*(*t*) is below the critical threshold U_*_ during the treatment.

The restriction that the toxicity due to the optimal therapy should be smaller or equal than the obtained by the standard treatment, means that

$$E_{\text{opt}}=E\left(N_{\text{opt}},d_{\text{opt}}\right)\leq E_{h}=E\left(N=30,d=1.8\text{Gy}\right),$$ (9)

with *E*_opt_ given by Eq (6). Thus, for every choice of *d* we choose the number of doses *N* to satisfy

$$N=\left\lfloor \frac{E_{h}}{\alpha_{h}}\frac{1}{d+\alpha_{h}d^{2}/\beta_{h}}\right\rfloor$$ (10)

In addition to the biologically motivated constraints, a minimum dose per fraction of 0.5 Gy was set to avoid situations involving an excessive number of RT sessions.

### Parameter estimation

Proliferation rates for LGGs (*ρ*) have been estimated to be around 0.003 day^−1^ in previous works [19, 22, 37, 40] which gives a doubling time of the order of one year. From the most indolent tumors to those more aggressive within the class of LGGs the full range may comprise an order of magnitude, that is from 0.002 day^−1^ to 0.01 day^−1^.

As to the cell density parameters *U*_0_ and U_*_, the normal brain tissue has low cellularity and the cell density leading to symptoms (*U*_0_) is probably dependent on the location of origin of the tumor. However, most LGGs are supratentorial and appear in the white matter. We will take the initial density *U*_0_ to be around 0.3 which means that symptoms arise when 30% of the space is occupied by tumor cells, a number well beyond the normal physiological value that may be around 10-15% (for non-pathological cells). We took the maximal tissue density leading to irreversible damage U_*_ to be around 0.5-0.65 [39] which corresponds to the maximal cell density that tissue is able to support without substantial changes to the microenvironment. Since both U_*_ and *U*_0_ may have a range of variation, we performed a study of the influence of these parameters on our results.

In addition, it is known that gliomas are radio-resistant tumors with high surviving fractions *in vitro* and mixed response to radiation *in vivo*. We will use here the approach of [37] that estimated the surviving fraction for doses of 1.8 Gy from the radiological response fitting typically observed dynamics of mean tumor diameters from [41]. This gives survival fractions *S*_*f*_(1.8) in the range of 0.8-0.9. Taking the values of the radiobiological parameters for LGG to be *α*_*t*_ = 0.0564Gy^−1^ and *β*_*t*_ = 0.0188 Gy^−2^ we get *S*_*f*_(1.8) = 0.85 and the ratio *α*_*t*_/*β*_*t*_ = 3Gy in agreement with the known values for this paramater. [42].

It is worth to remark that only the ratio *α*_*h*_/*β*_*h*_ is necessary to compute *E*_*h*_. The ratio for healthy brain tissue, *α*_*h*_/*β*_*h*_, is about 10 Gy [42]. Moreover, small variations of this choice did not affect our results.

The parameters used through this paper are summarized in (Table 1).

**Table 1:** Values of the biological and clinical parameters used in the mathematical model of LGG evolution.

Variable	Description	Value (units)	References
*ρ*	Proliferation rate	0.002-0.01 day^−1^	[37, 38, 40]
*d*	Dose per fraction	0.5-3.2 Gy	[42]
*S*_*f*_(1.8)	Survival fraction	0.85	[42]
*U*_0_	Initial cell density	0.1-0.3	[39]
U_*_	Critical cell density	0.5-0.65	[39]
*α*_*t*_/*β*_*t*_	LGG ratio	3 Gy	[42]
*α*_*t*_	Linear damage to LGG	0.0564 Gy^−1^	Estimated
*α*_*h*_/*β*_*h*_	Ratio for normal brain	10 Gy	[42]

## Results

### The time to malignant transformation depends on the fractionation scheme and tumor’s proliferation rate

**Fig 3:**
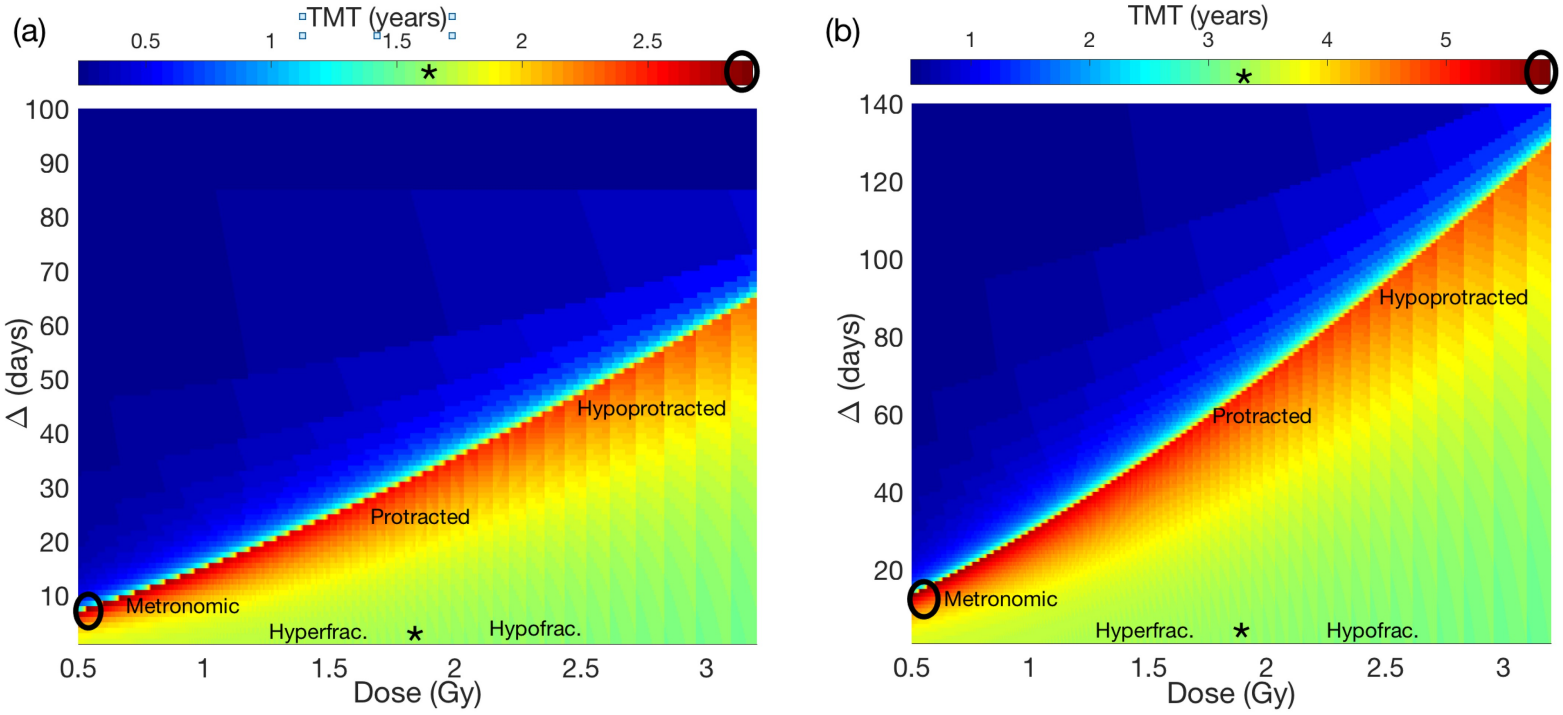
Results for the TMT under different fractionation schemes. Stars and circles indicate the location of the standard and optimal treatments respectively on the (Dose, Δ) plane and their associated TMT. (a) *ρ* = 0.01 day^−1^. Optimal fractionation is *d*_opt_ = 0.5 Gy every 6 days (Δ_opt_ = 6 days), and TMT = 2.9 years. (b) *ρ* = 0.005 day^−1^. Optimal fractionation is *d*_opt_ = 0.5 Gy and Δ_opt_ = 16 days TMT = 5.7 years.

Representative examples of our numerical simulations for the TMT under different fractionation schemes are shown in Fig 3. Specifically we present results for proliferation rates: *ρ* = 0.01 day^−1^ (fast-growing tumor, Fig 3(a)) and *ρ* = 0.005 day^−1^ (slowly-growing tumor, Fig 3(b)).

For every *ρ* in the range 0.002-0.01 day^−1^, we obtained that choosing the optimal dose, *d*_opt_, to be equal to 0.5 Gy, and the optimal time between fractions (Δ_opt_) being more than one day, led to the longest TMT. Thus, metronomic scheduling was found to be the optimal one for every proliferation value, these results can be observed in Fig 3. For fast-growing LGGs with *ρ* = 0.01 day^−1^, the optimal inter-spacing (Δ_opt_) was found to be 6 days, Fig 3(a). For slowly-growing LGGs with *ρ* = 0.005 day^−1^, Δ_opt_ = 16 days, Fig 3(b). These findings point to a dependence of the optimal time spacing between doses on the proliferation rate to be analyzed later in this section.

Hence, the optimal therapy would be the one with fixed dose equal to 0.5 Gy and its corresponding Δ_opt_.

The predicted TMT for the standard scheme is 1.6 years for fast-growing LGG typical parameters (*ρ* = 0.01 day^−1^) and 3.3 years for slowly growing LGG (*ρ* = 0.005 day^−1^). Using the optimal therapeutical schedulings led to TMT = 2.9 years (*ρ* = 0.01 day^−1^) and TMT = 5.7 years (*ρ* = 0.005 day^−1^) respectively. Thus providing prolonged survivals beyond 70% of the initial times.

Pérez-García and Pérez-Romasanta [39] predicted that protracted schemes would lead to a delay of the MT, which is consistent with our results (see Fig 3). Indeed, enlarging the time interval between doses was always beneficial when comparing to the standard therapy provided the interval is not larger than the corresponding Δ_opt_. On the basis of the results shown in Fig 3 it is clear that both protracted and hypoprotracted therapies delay malignant transformation although the largest gain was obtained for the metronomic schemes.

There is little or no advantage in using hyper or hypofractionated schemes. This is also consistent with the results by Galochkina et al [38], who found that small variations from the standard scheme did not result in significant delays of the TMT.

The choice of the time spacing between fractions Δ is critical in getting a substantial delay of the TMT. Indeed, if Δ is taken to be larger than the optimal one, Δ_opt_, the therapy becomes useless with very small survival gains (blue upper left regions in Fig 3(a) and 3(b)). In that situation, the tumor reaches the critical threshold, U_*_, before completing the treatment. So, in this case, the treatment is not able to maintain the tumor under control and would affect instead the faster growing HGG tumor. Thus although that choice would be unable to delay optimally the MT it would provide some therapeutical benefit of the order of the one currently obtained when treating trasformed HGG tumors.

### Optimal treatments control tumor growth below the cell density threshold for the MT

**Fig 4:**
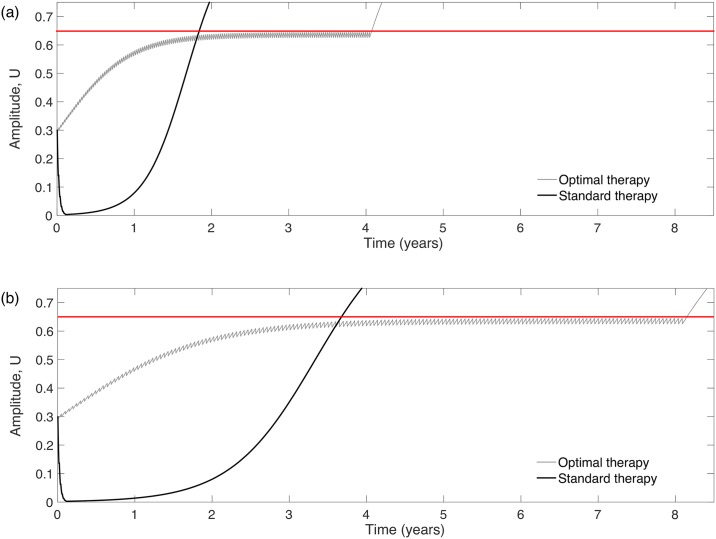
Evolution of the tumor amplitude under standard and optimal therapies (black and gray curves respectively). The value of the parameters used are: *U*_0_ = 0.3, U_*_ = 0.65 and (a) *ρ* = 0.01 day^−1^ (a fast-growing virtual LGG) and (b) *ρ* = 0.005 day^−1^ (a slowly growth virtual LGG).

Fig 4 compares the tumor amplitude (maximum cell density) evolution for the standard versus the optimal fractionations for (a) a fast growing and (b) a slowly growing LGG. The variations observed in the TMT can be explained by Eq (7). The larger the time interval between doses is, the longer the TMT.

Standard fractionation leads to a substantial initial decrease of the amplitude and a subsequent tumor regrowth. Metronomic therapies instead intend to maintain the tumor cell density under the critical threshold for as long as the treatment can be maintained. Thus, the standard therapy is more efficient in reducing the total tumor load for a shorter period of time. However, a complete elimination of the tumor load is known to be unlikely due to the radio resistance of glioma cells.

### TMT using optimal therapies does not depend on the initial cell density *U*_0_, but on the critical threshold density U_*_

**Fig 5:**
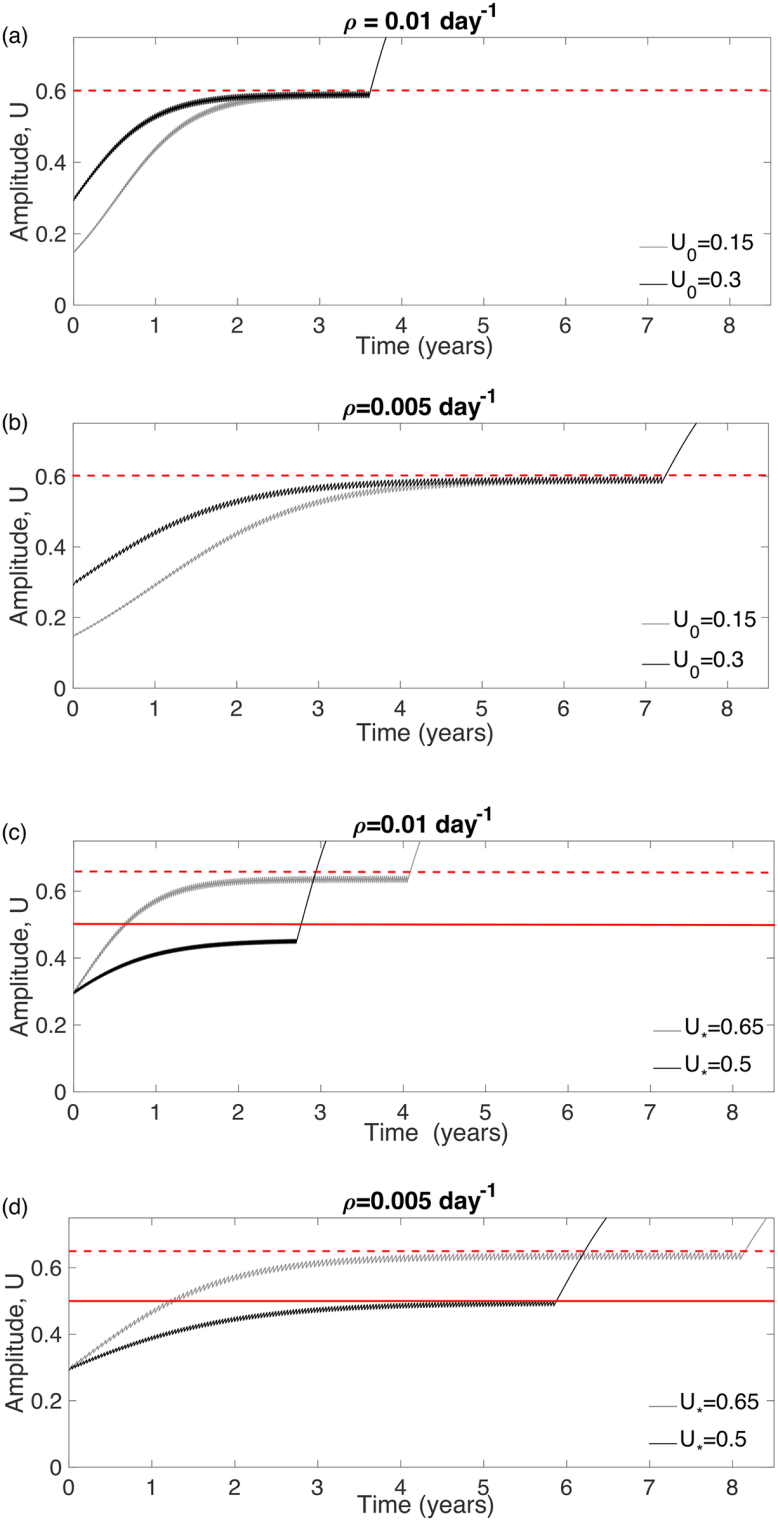
Tumor amplitude evolution for eight virtual tumors under the effect of the optimal radiation treatment. (a) *ρ* = 0.01 day^−1^, U_*_ = 0.6. (b) *ρ* = 0.005 day^−1^, U_*_ = 0.6. (c) *ρ* = 0.01 day^−1^, *U*_0_ = 0.3. (d) *ρ* = 0.005 day^−1^, *U*_0_ = 0.3. (a-b) Show the comparison between two simulations with *U*_0_ = 0.15 and *U*_0_ = 0.3 under optimal therapies. (c-d) Show the comparison between two simulations with U_*_ = 0.5 and U_*_ = 0.65 under optimal therapies.

We studied the impact of the initial cell density, *U*_0_ and the critical threshold, U_*_, on the TMT under the optimal fractionation scheme. To do so, we chose four different sets of parameters (*U*_0_, U_*_) as follows: First, we fixed U_*_ and chose two different *U*_0_ values and, then, we fixed *U*_0_ and chose two different U_*_ values. For each set, we performed two simulations, one for *ρ* = 0.01 day^−1^ and, other for *ρ* = 0.005 day^−1^. These results are summarized in Fig 5.

In our simulations, the initial tumor density did not have a substantial effect on the TMT under optimal therapies (cf. Fig 5(a) and 5(b)). However, the critical density U_*_ had a strong influence on the TMT (cf. Fig 5(c) and 5(d)).

**Fig 6:**
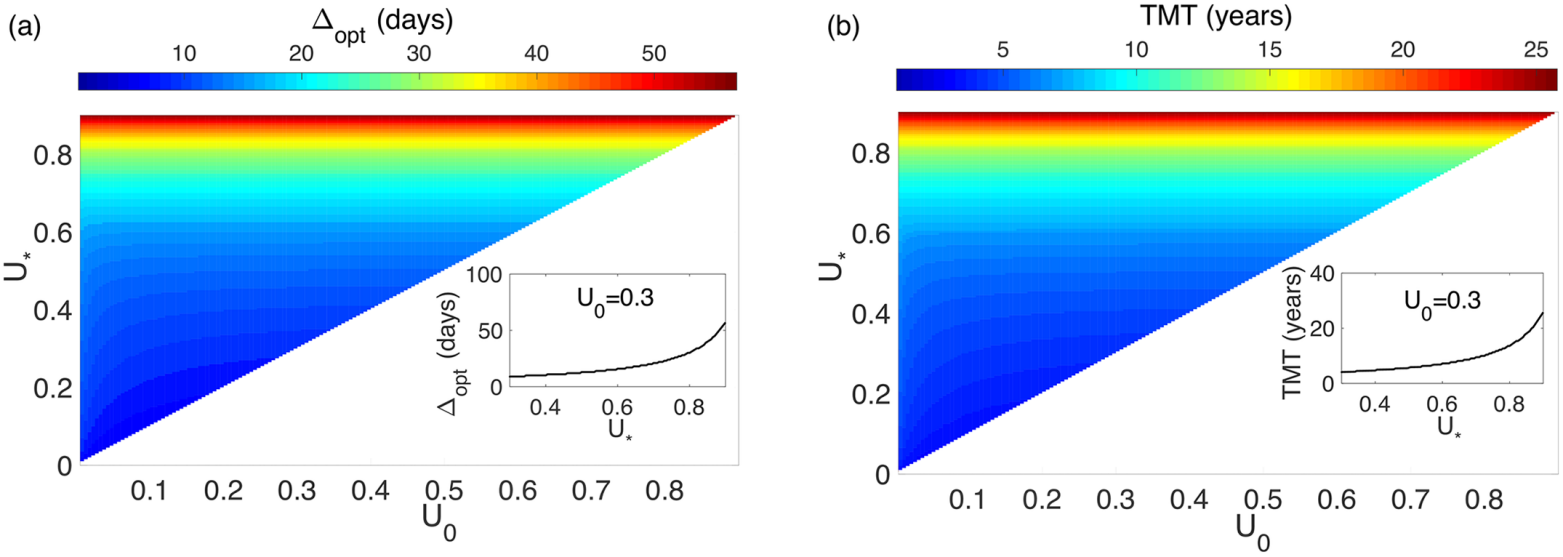
Dependence of the optimal Δ, (Δ_opt_) and TMT on the initial and critical tumor cell densities for *ρ* = 0.005 day^−1^. (a) Δ_opt_ as a function of *U*_0_ and U_*_. (b) TMT computed using the optimal Δ_opt_(*U*_0_, U_*_). The insets show the curves for *U*_0_ = 0.3.

A study of the full parameter space Δ_opt_(*U*_0_, U_*_), TMT(*U*_0_, U_*_) for several values of *ρ* was performed to confirm the results. Fig 6 presents an example that confirms the previous findings. Both the optimal fractionation and TMT where independent of *U*_0_ but dependent on U_*_.

**Fig 7:**
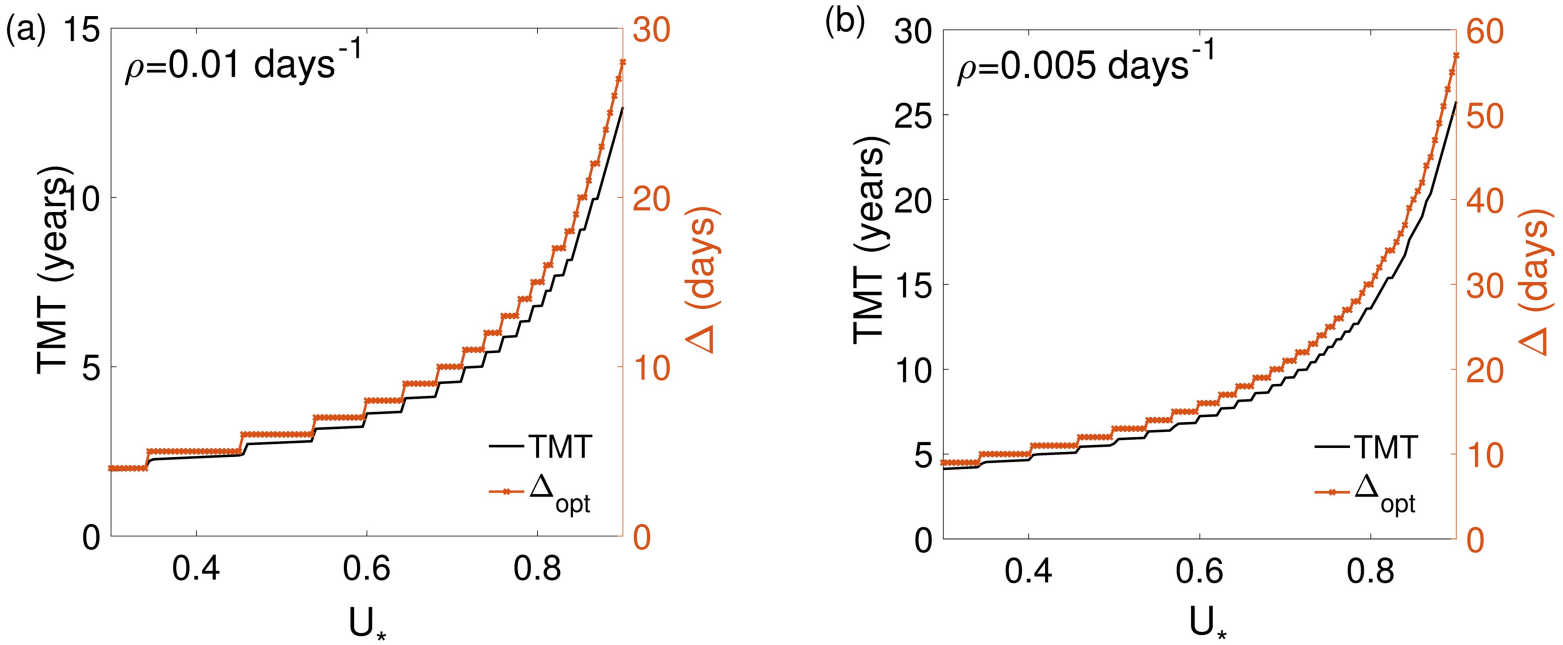
Dependence of the TMT and Δ_opt_ on U_*_ for *U*_0_ = 0.3. (a) *ρ* = 0.01 day^−1^, (b) *ρ* = 0.005 day^−1^. In both cases, the optimal fractionation for each parameter set was used.

Fig 7 shows the dependence of the optimal protocol (Δ_opt_) on U_*_ for two different proliferation values. The dependence on U_*_ of the fractionation parameters was also linked to the proliferation rate. In Fig 7 we plotted the TMT and the time between fractions as a function of the threshold, U_*_.

### Metronomic therapies provide substantial survival advantages for virtual patients

In order to quantify the delay in TMT obtained from the optimal metronomic fractionations we performed several series of simulations. First, we fixed *U*_0_ = 0.3 and U_*_ = 0.5 as discussed in previous works [39] and computed Δ_opt_ and the corresponding TMT as a function of *ρ*. This was done for both the optimal parameters and the standard fractionation (*N* = 30, Δ = 1 and *d* = 1.8Gy).

**Fig 8:**
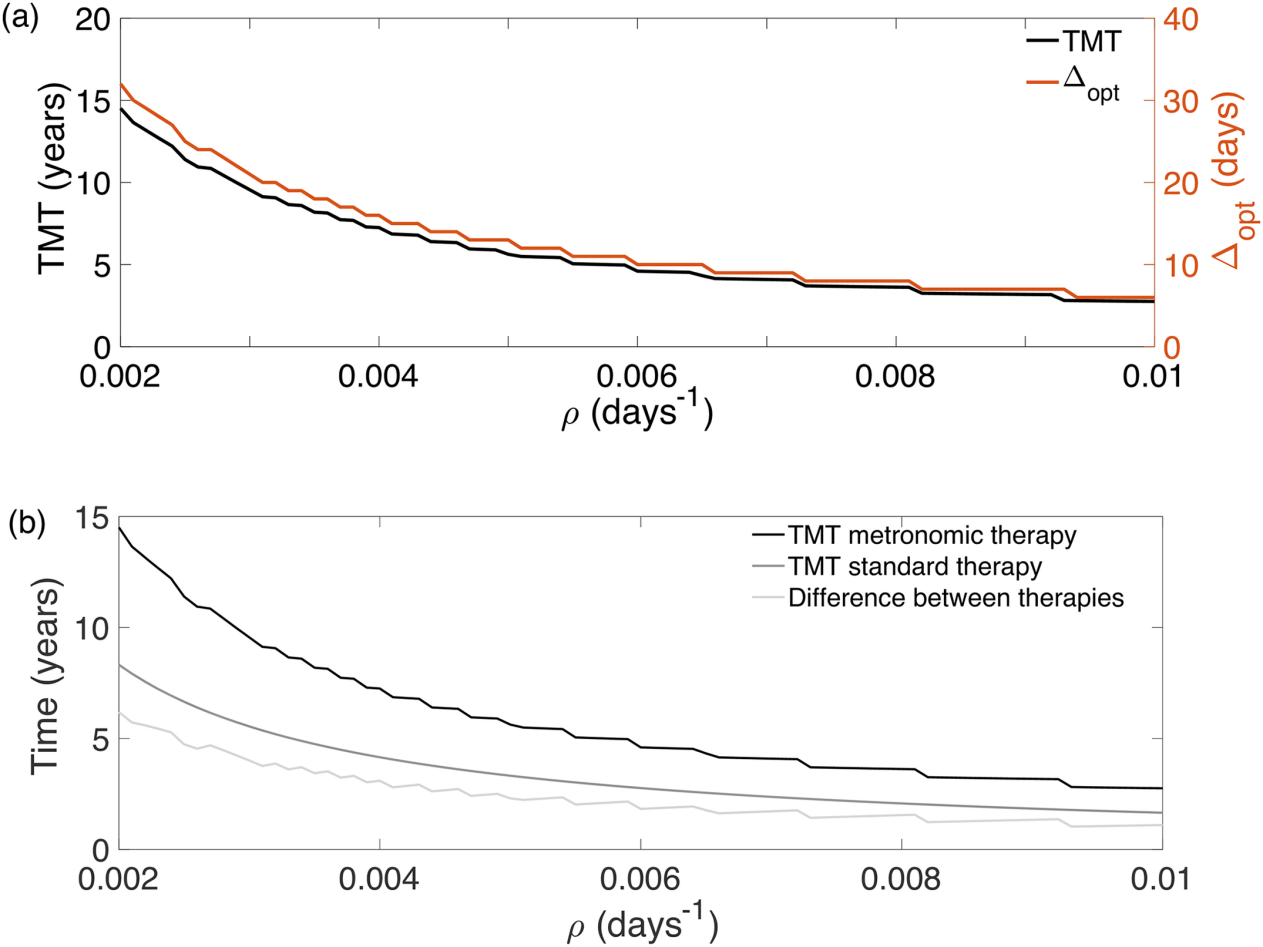
The optimal protocol delays substantially the MT considering the optimal time between fractions Δ_opt_ for *U*_0_ = 0.3, U_*_ = 0.5 and *ρ* ∈ [0.002, 0.01]. (a) Δ_opt_ and TMT obtained with the optimal protocol. (b) TMT for both the optimal (black curve) and the standard protocols (dark gray curve). The light gray curve represents the differences between their TMTs. The later provides a quantification of the benefit obtained from the optimal fractionation over the standard one.

The results for Δ and TMT are shown in Fig 8(a). The comparison between the TMT for the standard and optimal fractionation and the difference between them are shown in Fig 8(b). The time delay of the malignant transformation is substantial and even of the order of several years for slowly growing tumors. In general, the TMT delay was found to be larger in absolute numbers for slowly growing tumors but it was always very substantial in percent terms (around 80-90%) for fast growing LGGs.

### Protracted and hypoprotracted schemes delay substantially the malignant transformation

**Fig 9:**
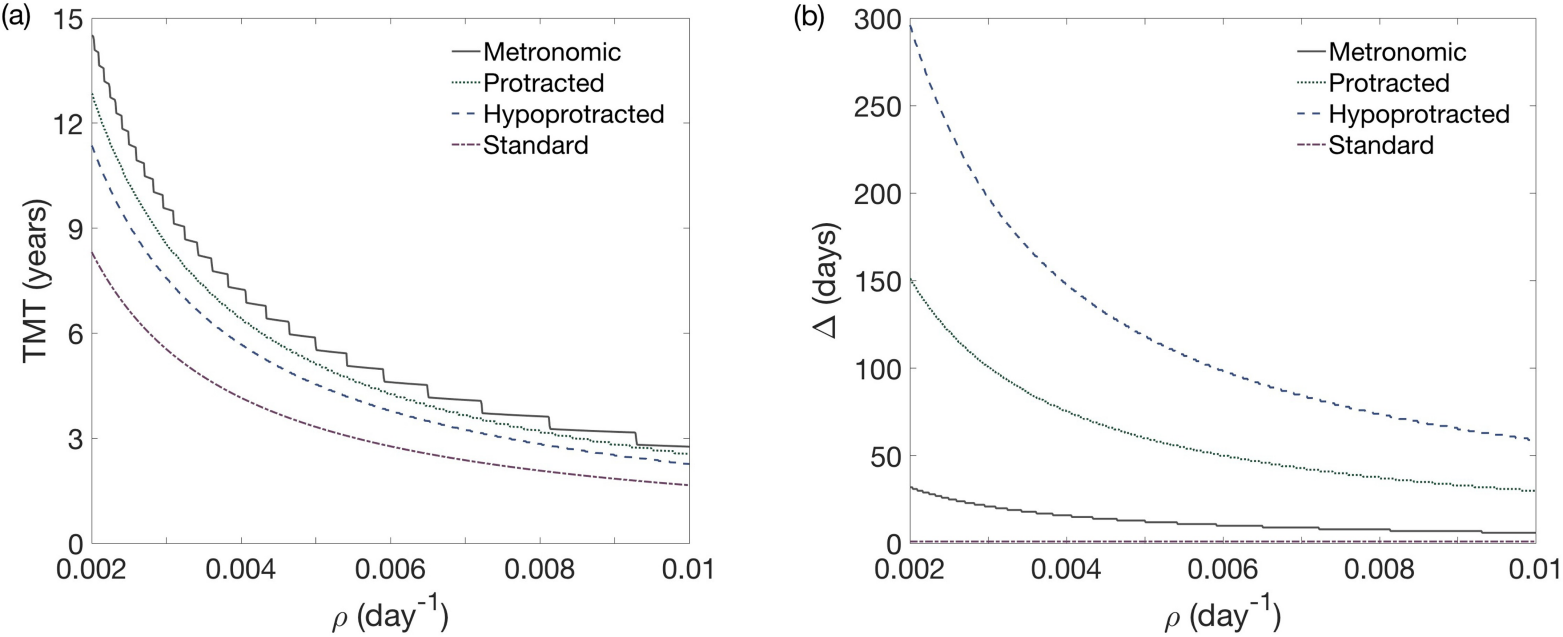
Comparison of four different fractionation schemes: Optimal fractionation (black line), best protracted scheme obtained for *d* = 1.8 Gy (grey line), best hypoprotracted treatment obtained with *d* = 3.2 Gy (light grey) and standard fractionation (dashed line). In all cases the range 0.002 < *ρ* < 0.01 was studied. Pannel (a) shows the TMT as a function of *ρ* and (b) the value of Δ used for each of the schemes.

Although hypoprotracted and protracted schemes are suboptimal therapies, they substantially delayed the malignant transformation in-silico from one year (for fast-growing tumors) to three years (for slowly-growing tumors). Some examples are shown in Fig 9. Thus, large parameter regions in the plane (Δ, *d*) provide therapeutical schemes better than the standard one. In many cases the TMTs obtained are close to the optimal ones (Fig 9).

### A conservative suboptimal scheme improves standard treatment results for any proliferation rate

If it were possible to know the specific parameters *ρ* and U_*_ for each patient our model could suggest the best personalized optimal therapy. However, this is a very challenging task. Thus, it may be preferable to find a suboptimal RT scheme improving the standard treatment results for all LGG patients.

**Fig 10:**
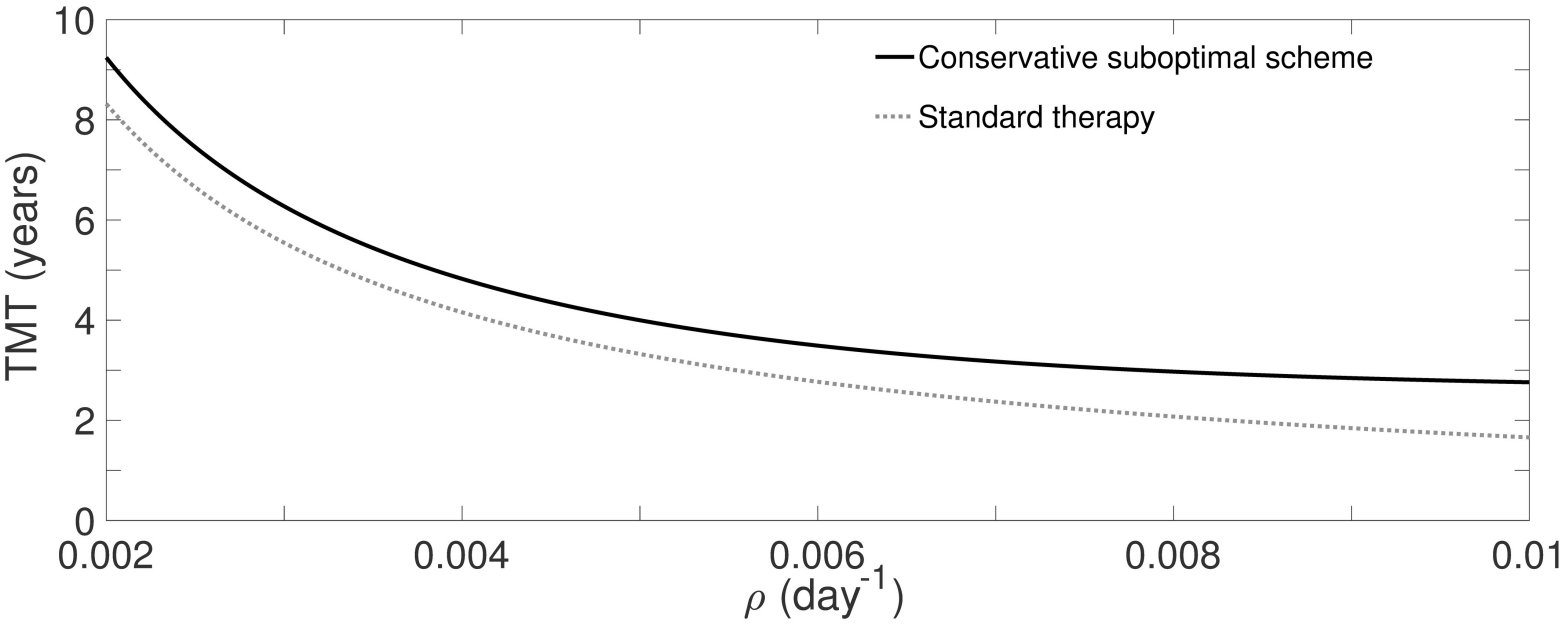
Benefit quantification of the conservative suboptimal treatment, Δ_subopt_ = 6 days, against the standard one for *ρ* ∈ [0.002, 0.01].

A possibility was found by using the optimal treatment for fast-growing LGGs, specifically the one presented in Fig 3(a) (*d*_opt_ = 0.5Gy and Δ_opt_ = 6 days). Fig 10 shows that the new scheme improves the results of the standard one for any value of 0.002 ≤ *ρ* ≤ 0.01.

The benefit, in terms of the time delay of the MT, was always larger than a year for every virtual tumor. For fast-growing LGGs, this scheme is close to the optimal one. For all the suboptimal treatment simulations, it was ensured finishing the treatment before MT occurs, with same or lower toxicity than the standard one.

## Discussion

Mathematical modeling has the potential to help in selecting LGGs patients who may benefit from RT and in developing specific optimal fractionation schemes for selected patient subgroups. Many papers have developed mathematical models of response of gliomas to RT [31–38, 40]. However, the mathematical study of what is the optimal RT course for LGGs has received little attention [39].

Our results show that a substantial delay of the MT could be achieved by using non-standard fractionation regimes such as metronomic, protracted or hypoprotracted schemes. In all cases the toxicity of the usual scheduling computed using the LQ model was preserved. Metronomic fractionations were found to be the ones delaying the most the MT. These schemes were also robust leading to survival gains when the time between RT fractions was chosen to be sub-optimal.

In order to limit the total number of doses to a reasonable limit the minimal dose per fraction was fixed to be 0.5 Gy. However, the optimal inter-spacing time was dependent on the tumor growth rate: being 6 days for fast-growing LGGs and 16 days for slowly-growing LGG.

Intuitively what the optimization scheme does is to let the tumor grow in size and treat it when it is close to the transformation density. Thus it is based on the idea that larger tumors have slower growth rates. This strategy may be useful for this kind of slowly-growing tumors that do not metastatize. In contrast, classical intensive treatment strategies take the tumor to a dynamical region where it regrows faster because of the accelerated repopulation phenomenon (i.e. the exponential growth of the logistic equation for small densities).

In addition to this phenomenon there is another one not accounted for in our paper. Not only the tumor grows slowly as a whole but also very few cells are proliferating in a LGG. Thus intensive therapies target only a small subset of the tumor’s population that is active for the 6 weeks of treatment. Fractionation schemes with fractions well-separated in time could target different tumor cells subpopulations and be substantially more effective what would add an extra improvement to the results found in this paper.

It is interesting that, in our in-silico approach, both fast- and slowly-growing LGG benefited from the metronomic therapy although slowly-growing LGGs experienced a larger advantage. For fast-growing LGGs, suffering the transformation earlier, the delay in MT was always found to be larger than one year. For slowly-growing LGGs the survival benefit was substantial, even of six years for the smallest values of the proliferation rate used, *ρ* = 0.002 day^−1^.

Some delay in the MT can be expected by choosing a conservative scheduling (i.e. applying the optimal strategy for fast-growing LGGs to all tumors). Tailoring the treatment to individual patients requires having some information on the tumor growth rate. It was proposed that a way of getting that information would be to probe the LGG with a small amount of RT [37]. A different possibility would be to use data from pathology, such as Ki67 or MIB-1 labeling indexes when available. However, this information is dependent on the tumor sample taken and may lead to a proliferation rate underestimation. Also since the decision on using RT is taken some time after the surgery there is no guarantee that original proliferation indexes would be representative of the tumor biology.

Since knowing the exact tumor behavior and parameters for each patient may be beyond reach with current technology, a personalized optimal therapy may be complicated nowadays. However, a conservative suboptimal scheme might provide at least an extra year delay to MT compared to the standard treatment for all patients. This suggested scheme is optimal for patients with fast-growing LGG tumors and consists of the use of low doses (*d* = 0.5 Gy) and distances between doses of Δ = 6 days. All virtual patients treated with the conservative suboptimal scheme finished the treatment before malignant transformation and obtained same or lower toxicity than the patients that received the standard RT care.

Protracted therapies seem to be more effective than hypoprotracted ones for all virtual tumors but hypoprotracted therapies considerably reduce the number of doses per patient. This has two practical advantages: (i) patients attend the hospital less times allowing them to follow their normal life, (ii) RT machines can be more available allowing the clinicians to attend more patients. The disadvantage is that if the inter spacing between doses is big enough, it may be necessary to recalculate the RT plans for each patient.

From a practical point of view, the increase in the number of patient’s visits to the hospital to receive a large number of RT sessions, as required by metronomic schedules, poses practical problems. The obvious first one is the personal inconvenience of getting to the hospital every week or every other week for extended periods of time, which would substantially increase the cost of treatment. However, given that LGGs are not frequent diseases and the large potential survival gains, the costs should be balanced with the treatment potential advantages.

The fact that low-dose therapies maintained for long times are found to be optimal raises the question of the potential use of brachytherapy beads to treat gliomas. In fact, some limited experiences point to very satisfactory results of iodine-125 in children with inoperable gliomas [49], LGG located in the central sulcus region [50] or large LGG patient cohorts [51] although prospective randomized studies are missing. Other studies reported results similar to those obtained with external beam RT [52]. Brachytherapy implies a decaying dose, typically of exponential form *d*(*t*) and thus requires a separate mathematical modeling. We plan to perform the analysis in the future.

Another implication of our work is that therapies that increase the critical cell density U_*_ might provide a benefit when combined with cytotoxic therapies such as RT. Since malignant transformation seems to be related to the development of hypoxia, necrosis and changes on tumor vasculature, we hypothesize that therapies targeting thrombotic events [24] could increase U_*_ and provide a synergistic benefit when combined with the optimal strategies discussed here.

Also antiangiogenic therapies may be helpful to defer the MT once the critical density is reached by blocking the development of tumor vessels. Indeed, it has been reported that the combination of bevacizumab + irinotecan appears to produce sustained disease control in some children with recurrent LGGs [53].

Finally, our findings may be also applicable to chemotherapy treatment, since despite the different ways of killing the cells, cytotoxic therapies such as the standard chemotherapeutical drug for LGGs, temozolomide, mainly damages DNA during mitosis. Indeed a model based on previous ideas for RT [39] has been recently developed providing an excellent description of the response of individual LGG patients to temozolomide [54]. Recent observations using vinblastine with a limited number of LGG patients may be related to our analysis [55], their observations suggest the use of a metronomic chemotherapy with weekly vinblastine after an induction by irinotecan-bevacizumab in order to improve progression-free survival in children with LGG.

## Conclusion

The goal of our study was to develop a mathematical methodology allowing to find the parameters of the radiation protocol delaying the most the TMT of LGGs.

The optimal fractionation scheme was found to be a metronomic one in all the situations studied where small fractions of radiations (in our case the minimal allowed one was d = 0.5 Gy) with moderate separations between fractions of several days were given for very long periods of time. The choice of metronomic schemes was shown to potentially delay the malignant transformation for variable times ranging from 1 to 6 years depending on the tumor’s growth parameters. The time between fractions was also found to depend strongly on the proliferation rate of each virtual tumor.

We studied also other types of fractionations which might be more applicable in clinical practice, such as protracted or hypoprotracted schemes. They were also found to provide a substantial delay of the time to the malignant transformation when compared with standard schemes.
